# Immobilization of *Thermoplasma acidophilum* Glucose Dehydrogenase and Isocitrate Dehydrogenase Through Enzyme-Inorganic Hybrid Nanocrystal Formation

**DOI:** 10.1007/s00284-023-03577-6

**Published:** 2024-01-18

**Authors:** Shusuke Oshima, Yuri Oku, Kotchakorn T.sriwong, Yutaro Kimura, Tomoko Matsuda

**Affiliations:** 1 Department of Life Science and Technology, School of Life Science and Technology, Tokyo Institute of Technology, 4259 Nagatsuta-Cho, Midori-Ku, Yokohama, 226-8501 Japan; 2 Department of Chemistry and California Institute for Quantitative Bioscience, University of California, Berkeley, Berkeley, California 94720 USA

## Abstract

The development of green catalysts, specifically biocatalysts, is crucial for building a sustainable society. To enhance the versatility of biocatalysts, the immobilization of enzymes plays a vital role as it improves their recyclability and robustness. As target enzymes to immobilize, glucose dehydrogenases and carboxylases are particularly important among various kinds of enzymes due to their involvement in two significant reactions: regeneration of the reduced form of coenzyme required for various reactions, and carboxylation reactions utilizing CO_2_ as a substrate, respectively. In this study, we immobilized *Thermoplasma acidophilum* glucose dehydrogenase (*Ta*GDH) and *T. acidophilum* isocitrate dehydrogenase (*Ta*IDH) using a previously reported method involving the formation of enzyme-inorganic hybrid nanocrystals, in the course of our continuing study focusing on carboxylation catalyzed by the free form of *Ta*GDH and *Ta*IDH. Subsequently, we investigated the properties of the resulting immobilized enzymes. Our results indicate the successful immobilization of *Ta*GDH and *Ta*IDH through the formation of hybrid nanocrystals utilizing Mn^2+^. The immobilization process enhanced *Ta*IDH activity, up to 211%, while *Ta*GDH retained 71% of its original activity. Notably, the immobilized *Ta*GDH exhibited higher activity at temperatures exceeding 87 °C than the free *Ta*GDH. Moreover, these immobilized enzymes could be recycled. Finally, we successfully utilized the immobilized enzymes for the carboxylation of 2-ketoglutaric acid under 1 MPa CO_2_. In conclusion, this study represents the first immobilization of *Ta*GDH and *Ta*IDH using the hybrid nanocrystal forming method. Furthermore, we achieved significant activity enhancement of *Ta*IDH through immobilization and demonstrated the recyclability of the immobilized enzymes.

**Supplementary Information:**

The online version contains supplementary material available at 10.1007/s00284-023-03577-6.

## Introduction

Biocatalysts play an important role in the development of a sustainable society due to their environmentally friendly nature and several advantageous properties, including safety, chemo-, regio-, and enantioselectivity, and high efficiency. Consequently, they have found successful applications in various industries such as chemical [1, 2], pharmaceutical [3], and food [1]. To enhance the versatility of biocatalysts, immobilization has emerged as a crucial strategy [4–6]. Among various immobilization strategies, the method of forming enzyme-inorganic nanocrystals is particularly advantageous because it is simple and effective in achieving high activity [7–9], although producing a large amount of product by a flow system using immobilized enzymes prepared by this method is challenging [10]. With this method, the immobilized enzyme can be prepared by mixing a phosphate-buffered saline (PBS) containing the enzyme and a metal-ion solution, incubating the solution, and separating the precipitated immobilized enzyme by centrifugation. Thus, before enzyme immobilization, immobilization supports do not have to be prepared using complicated procedures or purchased at a high cost. Moreover, the resulting immobilized enzymes possibly have higher activities than their corresponding free enzymes; this is in contrast with the observation that the activity of the immobilized enzyme prepared by conventional methods may be lower than that of the corresponding free enzyme owing to the inactivation of the enzymes during the immobilization processes and mass transfer restrictions of the substrate and product between the bulk solvent and the enzymes’ active sites [4]. Therefore, various enzymes, including lipase [11], peroxidases [9, 12, 13], alcohol dehydrogenases [14, 15], aldehyde dehydrogenase [16], and Baeyer–Villiger monooxygenase [17], have been successfully immobilized using this method. However, to the best of our knowledge, the application of this immobilization method to glucose dehydrogenases, which are essential enzymes for expensive coenzyme recycling [18–20], and isocitrate dehydrogenases, which play a crucial role in carboxylation reactions [21, 22], remains unexplored. Therefore, in this study, we employed this method to immobilize *Thermoplasma acidophilum* glucose dehydrogenase (*Ta*GDH) and *T. acidophilum* isocitrate dehydrogenase (*Ta*IDH), which were previously used in their free form for a carboxylation reaction directly using CO_2_ as a substrate [22].

In our previous study, we selected enzymes from a thermophile for coenzyme recycling.

and carboxylation because of their high CO_2_ pressure resistance [22]. As shown in Fig. S1, *Ta*IDH catalyzes the carboxylation and reduction of 2-ketoglutaric acid. Coupling the *Ta*GDH-catalyzed reaction for cofactor regeneration from NADP^+^ to NADPH shifted the equilibrium of the carboxylation/decarboxylation reactions towards carboxylation. However, these enzymes were utilized in their free form, which made recycling impossible, necessitating further exploration of immobilization methods. Hence, in this study, we immobilized these enzymes using the method of forming enzyme-inorganic nanocrystals. We evaluated the activity and recyclability of the immobilized enzymes and used them for the carboxylation reaction using 1.0 MPa CO_2_.

## Materials and Methods

### Strains and Materials

Recombinant *E. coli* strains, Rosetta TM(DE3)pLysS-pET21b(+)-*Ta*GDH (*Ta*0897, KEGG) and BL21(DE3)pLysS-pET21b(+)-*Ta*IDH (*Ta*0117, KEGG) constructed in our previous study [22], were used. The reagents were purchased from commercial sources, including Nacalai Tesque (Japan), Wako Pure Chemical Industries (Japan), Tokyo Chemical Industry Co. (Japan), Sigma-Aldrich (USA), and Bio-Rad (USA), and were used without further purification. Scanning electron microscope (SEM) analysis was conducted using a benchtop scanning electron microscope proX from Phenom-World (Netherlands), as reported previously [15].

### Enzyme Preparation and Heat Treatment [22]

*Ta*GDH and *Ta*IDH were prepared as reported previously. *Ta*GDH was prepared as follows. A single colony of the recombinant Rosetta TM(DE3)pLysS-pET21b(+)-*Ta*GDH cells was inoculated overnight in LB medium (5.0 mL) with carbenicillin (125 μg/mL) and chloramphenicol (20 µg/mL) at 250 rpm at 37 °C. The pre-cultured cells (250 µL) were transferred into LB medium (250 mL) with carbenicillin (125 μg/mL) and cultured at 250 rpm at 37 °C for 1 d. Isopropyl β-D-1-thiogalactopyranoside (IPTG) (100 µM) was then added, and the cells were cultured at 250 rpm at 37 °C for 1 d. The cells were harvested by centrifugation at 8000×*g* for 10 min at 4 °C, suspended in NaCl (10 mL, 0.85%(w/v)), collected by centrifugation at 8,000 × g for 10 min at 4 °C, and suspended in potassium phosphate buffer (10 mL, pH 7.0, 100 mM) containing phenylmethylsulfonyl fluoride (PMSF) (1 mM) and 1,4-dithiothreitol (DTT) (1 mM). The mixture was sonicated at 100 W for 30 min at 0 °C and centrifuged at 12,000×*g* for 30 min at 4 °C. The supernatant was treated at 60 ℃ for 15 min and centrifuged at 12,000×*g* for 30 min at 4 °C to obtain a partially purified enzyme. The supernatant was diluted to 1 U/mL with potassium phosphate buffer (pH 7.0, 100 mM) and used for further experiments (554 U, 145 mg).

*Ta*IDH was prepared using BL21(DE3)pLysS-pET21b(+)-*Ta*IDH with the same method as for the *Ta*GDH preparation shown above except for the use of only carbenicillin (125 μg/mL) to obtain a partially purified enzyme (229 U, 153 mg).

### Enzyme Immobilization [15–17]

The partially purified *Ta*GDH and *Ta*IDH, prepared as shown above, were immobilized using a similar method to our previous studies. Phosphate-buffered saline (PBS) (10 mM) was prepared by dissolving Na_2_HPO_4_ (final concentration: 10 mM) and KH_2_PO_4_ (final concentration: 1.8 mM) in distilled water and adjusting the pH using HCl(aq) or NaOH(aq). NaCl (final concentration: 137 mM) and KCl (final concentration: 2.7 mM) were then added. The amounts of only Na_2_HPO_4_ and KH_2_PO_4_ were varied to prepare the different concentrations of PBS. PBS containing the enzyme and metal-ion solutions (500 µL) was mixed by pipetting, incubated at 4 °C for 18 h, and centrifuged at 12,000 rpm for 5 min at 4 °C. The residual protein concentration in the supernatant (protein leakage) was determined using the Bradford method [23] to calculate the immobilization yield using Eq. (1).

1 $${\text{Immobilization}}\,{\text{yield}}\,\left( {{\% }} \right){\text{ = }}\frac{{\left[ {{\text{Protein}}\,{\text{concentration}}} \right]_{{\text{I}}} {\text{ - }}\left[ {{\text{Protein}}\,{\text{concentration}}} \right]_{{\text{R}}} }}{{\left[ {{\text{Protein}}\,{\text{concentration}}} \right]_{{\text{I}}} }} \times 100$$

where [Protein]_I_ = Initial protein concentration before immobilization (mg/mL) and [Protein]_R_ = Protein concentration remaining in the supernatant after nanocrystal (immobilized enzyme) formation and centrifugation (mg/mL).

The precipitant was suspended in distilled water, centrifuged at 12,000 rpm for 5 min at 4 °C, and suspended in PBS to a protein concentration of 0.25 mg/mL. The remaining activity was calculated using Eq. (2).

2 $${\text{Remaining activity}}\,\left( \% \right) = \frac{{{\text{Activity of immobilized enzyme }}\left( {\text{U}} \right)}}{{{\text{Activity of free enzyme before immobilization }}\left( {\text{U}} \right)}} \times 100$$

Further details for conditions for the enzyme immobilizations are found in Table S1.

### Activity Assay [22]

The activities of *Ta*GDH and *Ta*IDH were determined as described previously. All assays were performed in triplicate. The details for conditions for the assays are found in the supporting information.

#### Activity Assay of *Ta*GDH

HEPES–NaOH buffer (pH 6.5, 100 mM, 960 µL) and D-glucose (100 mM, 10 μL) were mixed and incubated at 37 °C for 15 min. NADP^+^ (10 mM, 20 μL) and enzyme (free enzyme (1 U/mL, 10 µL) or immobilized enzyme (0.25 mg/mL, 10 µL)) were then added. The initial reaction rate was determined by measuring the NADPH concentration at 340 nm for 2 min. One unit of enzyme activity was defined as micromoles of NADPH released by the oxidation of glucose per minute under the conditions mentioned above.

#### Decarboxylation and Oxidation Activity Assay of *Ta*IDH

HEPES–NaOH buffer (pH 6.5, 100 mM, 940 µL), DL-isocitric acid (10 mM, 10 µL), and MgCl_2_ (20 mM, 20 µL) were mixed and incubated for 15 min at 37 °C. NADP^+^ (10 mM, 20 µL) and enzyme (free enzyme (1 U/mL, 10 µL) or immobilized enzyme (0.1 mg/mL, 10 µL)) were then added. The initial reaction rate was determined by measuring the NADPH concentration at 340 nm for 3 min. One unit of enzyme activity was defined as micromoles of NADPH released by the decarboxylation and oxidation of DL-isocitric acid per minute under the conditions mentioned above.

### Carboxylation of 2-Ketoglutaric Acid and Determination of the Yield of Isocitric Acid [22]

The carboxylation reaction was conducted, and the yield of isocitric acid was determined as previously reported.

#### Carboxylation Reaction of 2-Ketoglutaric Acid

HEPES–NaOH buffer (pH 7.0, 1.0 M, 500 µL containing d-glucose (final concentration: 1.0 M), 2-ketoglutaric acid (final concentration: 20 mM), and MnCl_2_ (final concentration: 20 mM)), immobilized enzymes (*Ta*IDH: 0.50 U, *Ta*GDH: 0.032 U), and NADPH (10 mM, 50 µL (final concentration: 0.5 mM)) were added to a pressure-resistant vessel (10 mL). HEPES–NaOH buffer (1.0 M, pH 7.0) was then added for a total volume of 1.0 mL. CO_2_ was introduced until a pressure reached 1.0 MPa. The solution was stirred at 135 rpm for 30 min at 37 °C. The reaction was quenched by depressurization and the addition of a few drops of NaOH(s).

#### Determination of the Isocitric Acid Yield of the Enzymatic Carboxylation

The carboxylation yield (the concentration of isocitric acid in the product mixture) was determined using a previously reported enzymatic analysis method. Standard isocitric acid solutions were made by mixing HEPES–NaOH buffer (pH 7.0, 1.0 M containing d-glucose (final concentration: 1.0 M), MnCl_2_ (final concentration: 20 mM), and isocitric acid (final concentrations: 5.0, 7.5, 10, 15, and 20 mM)) and NADPH (10 mM, 25 µL). HEPES–NaOH buffer (1.0 M, pH 7.0) was added for a total volume of 0.5 mL, and a few drops of NaOH(s) were added. HEPES–NaOH buffer (1.0 M, pH 6.5, 960 µL), the quenched reaction mixture from the previous Section (10 µL) or a standard isocitric acid solution prepared above (10 µL), and partially purified *Ta*IDH (1 U/mL, 10 µL) were mixed and incubated for 15 min at 37 °C, and NADP^+^ (10 mM, 20 µL) was added. The initial reaction rate was determined by measuring the NADPH concentration at 340 nm. From the calibration curve obtained using the standard solutions, the concentration of the isocitric acid in the product mixture was calculated, and the reaction yield was determined using Eq. (3).

3 $${\text{Yield}}\,\left( \% \right) = \frac{{{\text{Concentration of isocitric acid after reaction}}}}{{{\text{Concentration of 2 - ketoglutaric acid before reaction}}}} \times 100$$

### Characterization of the Immobilized Enzymes by SEM Analysis [15]

SEM analysis was conducted as previously reported. The *Ta*GDH nanocrystals (*Ta*GDH: 0.25 mg/mL, pH 7.0 PBS: 7.5 mM, Mn^2+^: 5.0 mM, 18 h, 4 °C), *Ta*IDH nanocrystals (*Ta*IDH: 0.05 mg/mL, pH 7.0 PBS: 7.5 mM, Mn^2+^: 25 mM, 18 h, 4 °C), and the Mn_3_(PO_4_)_2_ crystals (pH 7.0 PBS: 7.5 mM, Mn^2+^: 25 mM, 18 h, 4 °C) as control were washed with distilled water several times before drying at room temperature. The dried nanocrystals were then used for SEM analysis.

### Statistical Analysis

Microsoft Excel was utilized for plotting and analyzing the data. The data were expressed as mean ± standard deviation of the triplicated activity assay. The *t* test tool in Microsoft Excel was used under the assumptions of equal variance, one-sides t-distribution, and hypothesized mean difference of zero to determine *P*-values to assess significant differences between means.

## Results

### Immobilization of *Ta*GDH

**Fig. 1:**
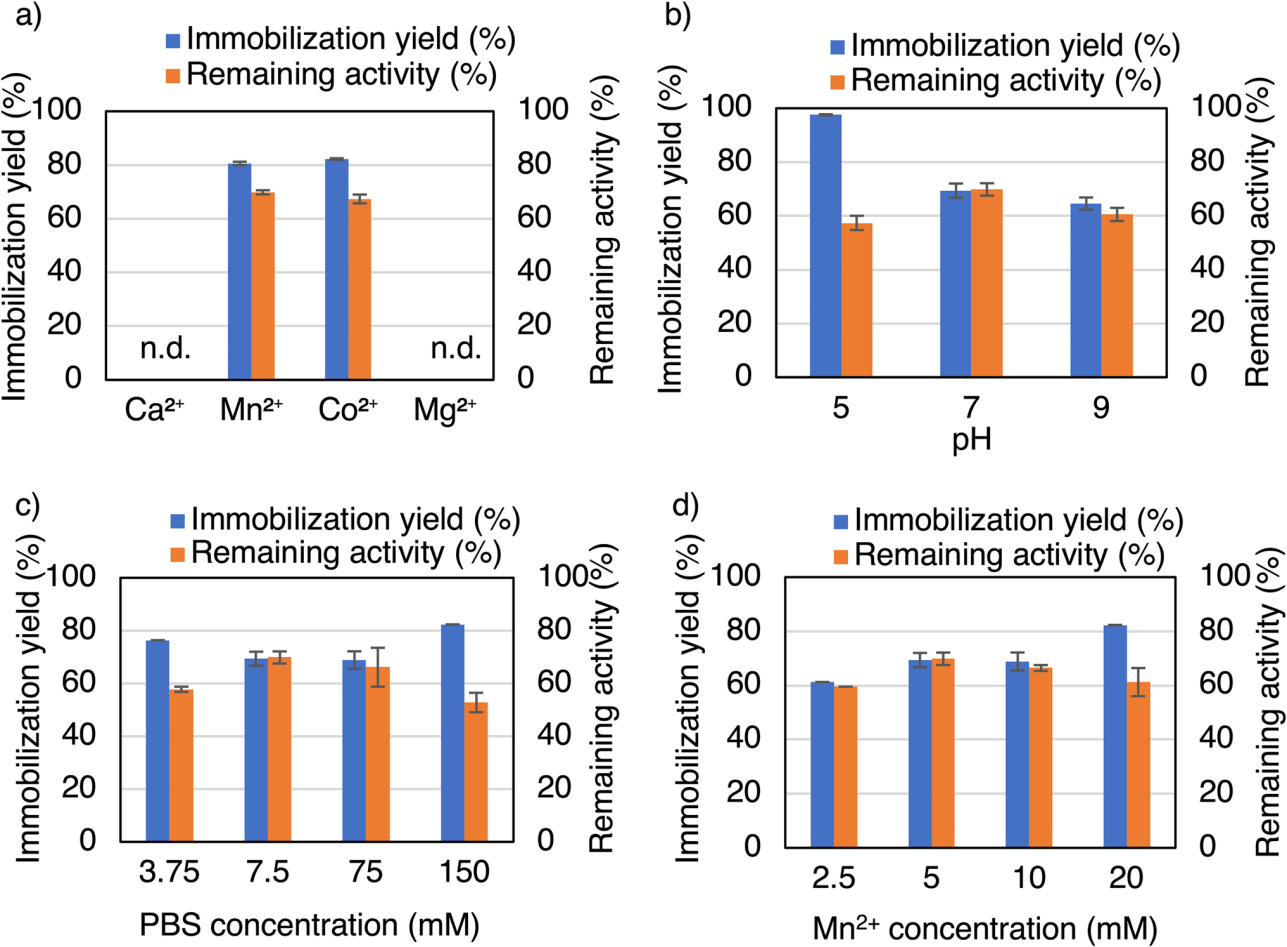
Effect of **a** metal ion, **b** pH, **c** PBS concentration, and **d** Mn^2+^ concentration on *Ta*GDH immobilization. After investigating the *Ta*GDH immobilization conditions, it was determined that pH 7.0, 7.5 mM of PBS, and 5.0 mM of Mn^2+^ were the most effective among the tested conditions. n.d. not detected. **b** *t* test to analyze the highest activity at pH 7.0: *P*-value (one-sided test) of pH 7.0 against pH 5: 0.020, pH 9: 0.038, **c** *t* test to analyze the high activity at 7.5 mM: *P*-value (one-sided test) of 7.5 mM against 3.75 mM: 0.011, 75 mM: 0.30, 150 mM: 0.012, **d** there was no significant difference between variables using *t* test

We first investigated the immobilization conditions for *Ta*GDH, followed by those for *Ta*IDH. Various metals such as Cu^2+^, Ca^2+^, Mn^2+^, Zn^2+^, Co^2+^, and Fe^2+^ can be used for nanocrystal formation [4, 7, 8]. Therefore, the metal ion type was first investigated for the immobilization of *Ta*GDH. CaCl_2_·2H_2_O, MnCl_2_·4H_2_O, CoCl_2_·6H_2_O, and MgSO_4_·7H_2_O were used in this study since the presence of these ions in the activity assay solution of free *Ta*GDH did not have a pronounced negative effect, as shown in Table S2. As shown in Fig. 1a, enzyme-inorganic nanocrystals were obtained using Mn^2+^ and Co^2+^ ions, and they did not precipitate when Ca^2+^ and Mg^2+^ ions were used. The subsequent experiments used Mn^2+^ ion because the carboxylation reaction yield using the free form of the enzymes was higher for the reaction using Mn^2+^ than using Co^2+^ [22].

**Fig. 2:**
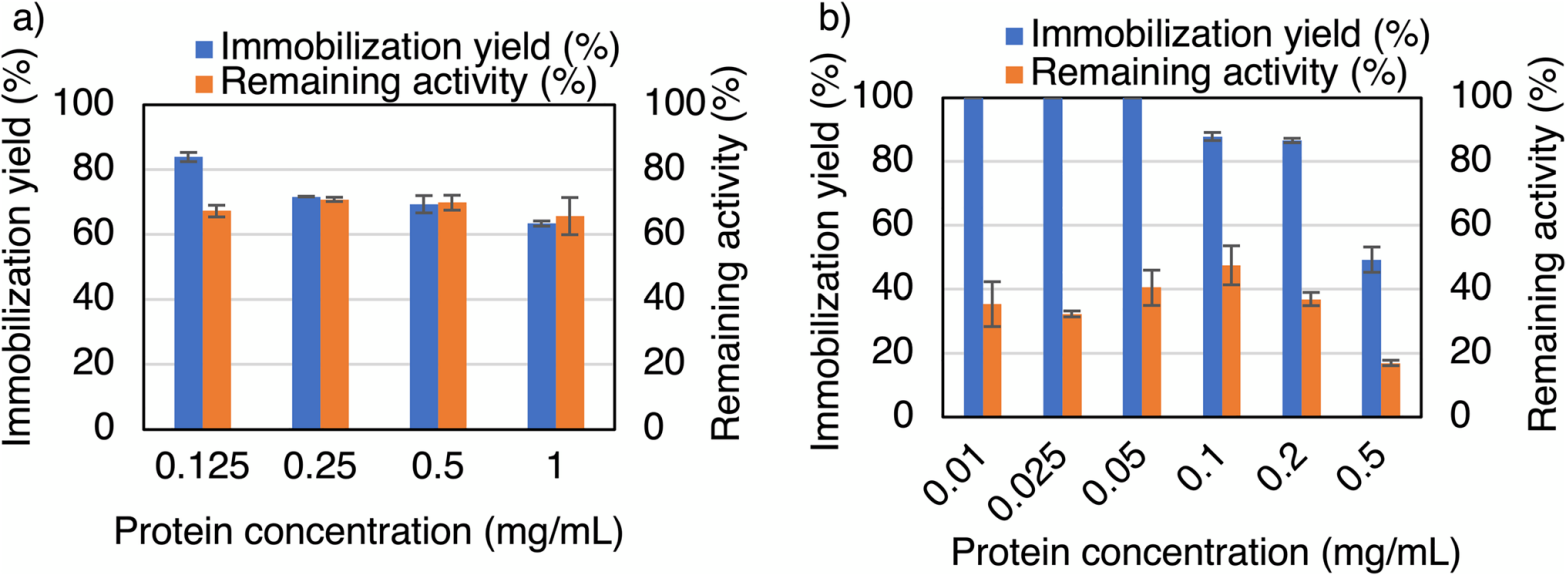
Effect of protein concentration **a** with 5 mM Mn^2+^ and **b** with 25 mM Mn^2+^ on *Ta*GDH immobilization. After investigating the *Ta*GDH immobilization conditions, we determined to employ the following conditions for the further study: 0.25 mg protein/mL with 5 mM Mn^2+^. **a** There was no significant difference between variables using *t* test. These conditions yielded an immobilization yield of 72% with a remaining activity of 71%

Next, the effects of pH, phosphate-buffered saline (PBS) concentration, Mn^2+^ concentration, and protein (enzyme) concentration on immobilization were investigated. The results are presented in Fig. 1b–d and Fig. 2a. As a result of these investigations, we determined to employ the following conditions for the further study: pH 7.0 and 7.5 mM of PBS, 5.0 mM of Mn^2+^, and 0.25 mg protein/mL, which gave an immobilization yield of 72% and remaining activity of 71%. Additionally, the effect of the protein (*Ta*GDH) concentration on immobilization was investigated at 25 mM Mn^2+^ (Fig. 2b), which is much higher than the previous Mn^2+^ concentration (5.0 mM Mn^2+^, Fig. 2a) because the optimum immobilization condition for *Ta*IDH was 25 mM Mn^2+^, as shown in next section. The optimum protein concentration was 0.1 mg/mL, resulting in an immobilization yield of 88% and remaining activity of 48%.

### Characterization of Immobilized *Ta*GDH

**Fig. 3:**
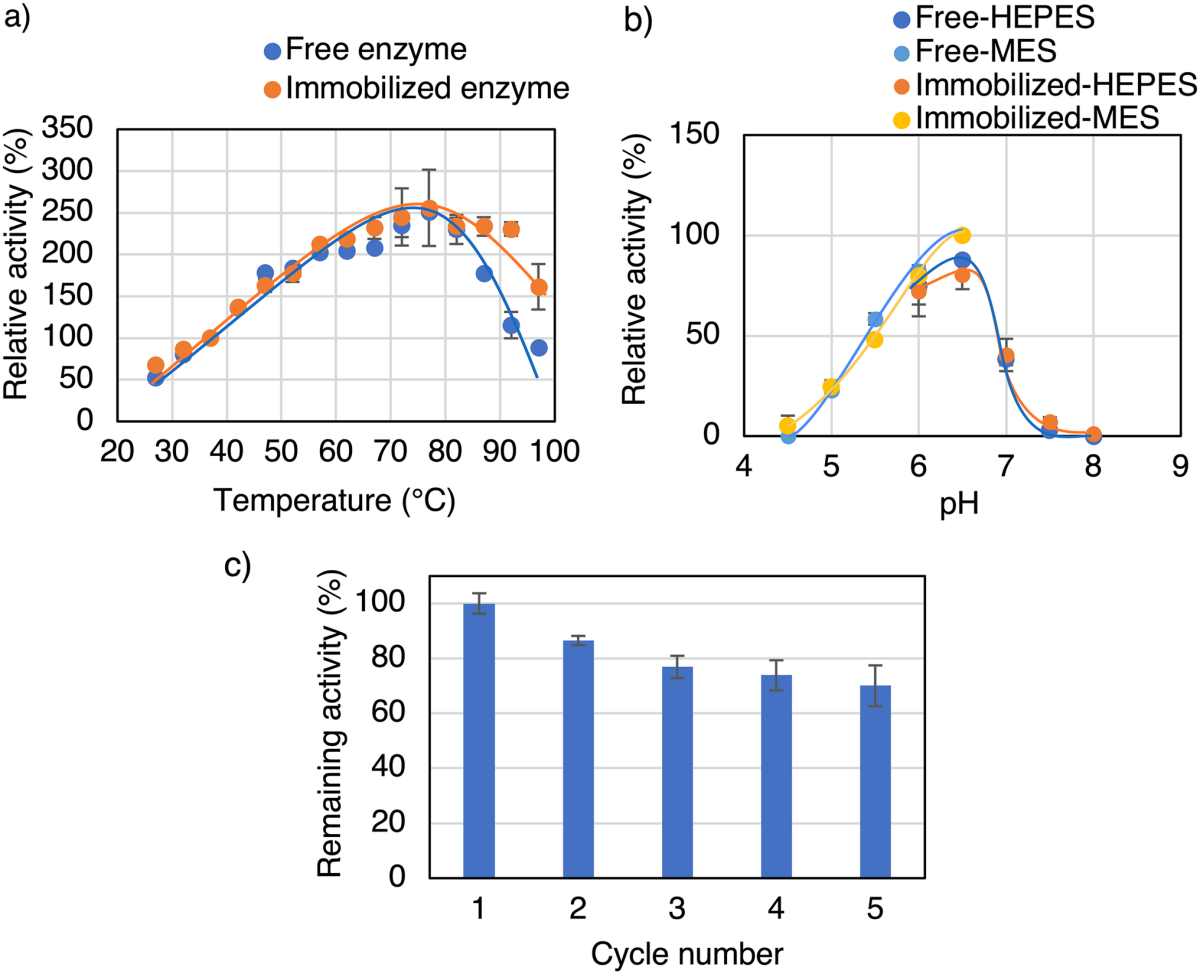
Effect of **a** temperature and **b** pH on the activity of free and immobilized *Ta*GDH and **c** recyclability of immobilized *Ta*GDH. Immobilization improved *Ta*GDH stability in the high-temperature region (*t* test to analyze the higher activity of the immobilized enzyme than the free enzyme at high temperature region (> 87 °C): *P*-value (one-sided test) of 77 °C: 0.29, 82 °C: 0.40, 87 °C: 0.0032, 92 °C: 0.0016, 97 °C: 0.019), but there was no significant effect of immobilization on optimum pH. The immobilized *Ta*GDH could be used up to five times, with a remaining activity of 70%

The immobilized *Ta*GDH synthesized under the optimal conditions (pH 7.0 and 7.5 mM PBS, 5.0 mM Mn^2+^, and 0.25 mg protein/mL) was characterized and compared with the free enzyme, and the morphology was examined by SEM analysis (Fig. S2b). Examination of the effect of temperature on the activity of free and immobilized *Ta*GDH revealed that the optimum temperature was 77 °C for both, and the activity above 87 °C was higher for the immobilized enzyme than for the free enzyme (Fig. 3a). Examination of the effect of pH on the activity of free and immobilized *Ta*GDH revealed that the optimum pH was 6.5 for both, and there was no significant difference between them (Fig. 3b). The recyclability of the *Ta*GDH nanocrystals was then examined. The *Ta*GDH nanocrystals could be used up to five times, with a remaining activity of 70% (Fig. 3c).

### Immobilization of *Ta*IDH

**Fig. 4:**
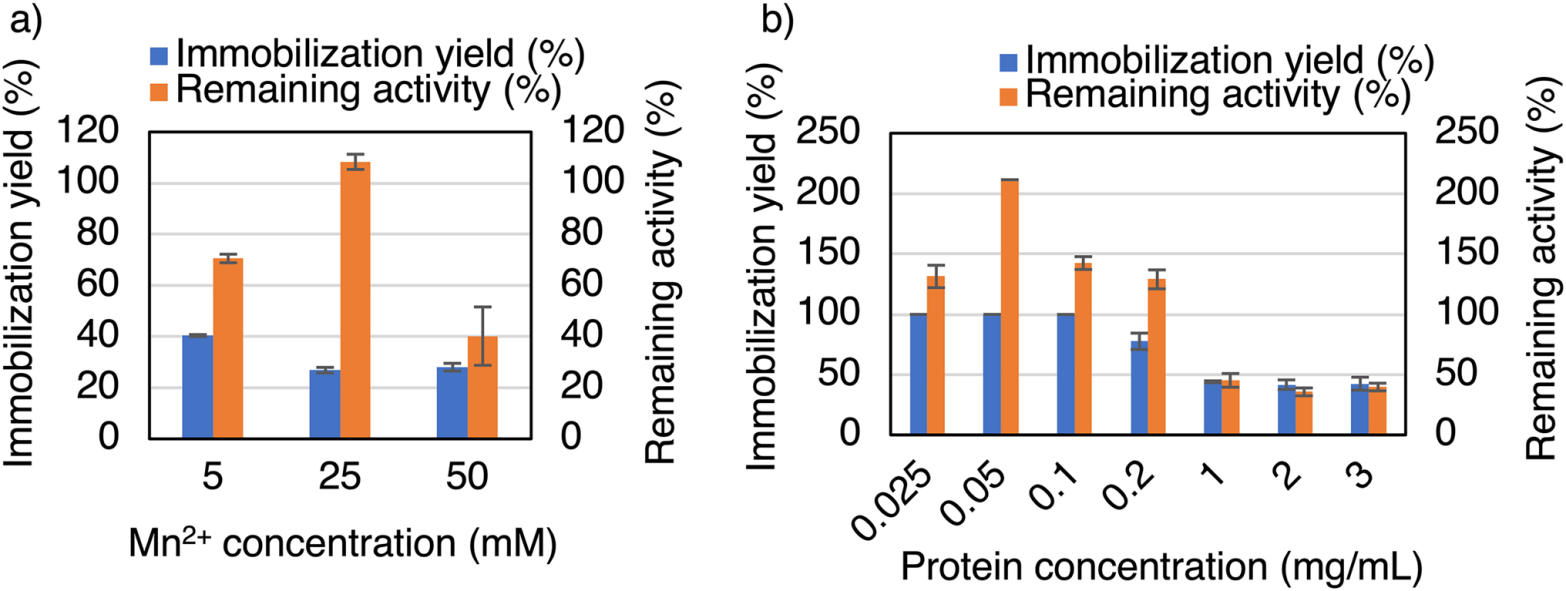
Effect of **a** Mn^2+^ concentration and **b** protein concentration on *Ta*IDH immobilization. After investigating the *Ta*IDH immobilization conditions using pH 7.0 and 7.5 mM of PBS, it was determined that 25 mM of Mn.^2+^ and 0.05 mg protein/mL were the most effective among the tested conditions. These conditions yielded an immobilization yield of > 99% with a remaining activity of 211%. **a** *t* test to analyze the highest activity at 25 mM: *P*-value (one-sided test) of 25 mM against 5.0 mM: 0.0040, 50 mM: 0.014), **b** *t* test to analyze that remaining activity is more than 100% at the concentration between 0.025 and 0.2 mg/mL: *P*-value (one-sided test) of 0.025 mg/mL: 0.030, 0.05 mg/mL: 0.000018, 0.1 mg/mL: 0.019, 0.2 mg/mL: 0.012)

The immobilization conditions for *Ta*IDH were also investigated. Because the optimum metal and PBS pH and concentration for *Ta*GDH immobilization were Mn^2+^ and pH 7.0 and 7.5 mM, respectively, the type of metal ion to be investigated was limited to Mn^2+^, and the PBS was fixed at pH 7.0 and 7.5 mM. First, the Mn^2+^ concentration was examined for *Ta*IDH immobilization, which resulted in an optimal concentration of 25 mM (Fig. 4a). The effect of protein concentration in 25 mM Mn^2+^ and pH 7.0 and 7.5 mM PBS was examined, achieving > 99% immobilization yield and 211% remaining activity at a protein concentration of 0.05 mg/mL (Fig. 4b). When the protein concentrations were varied between 0.025 mg/mL to 0.2 mg/mL, the remaining activity was also higher than the corresponding original free enzyme (> 100%).

### Characterization of Immobilized *Ta*IDH

**Fig. 5:**
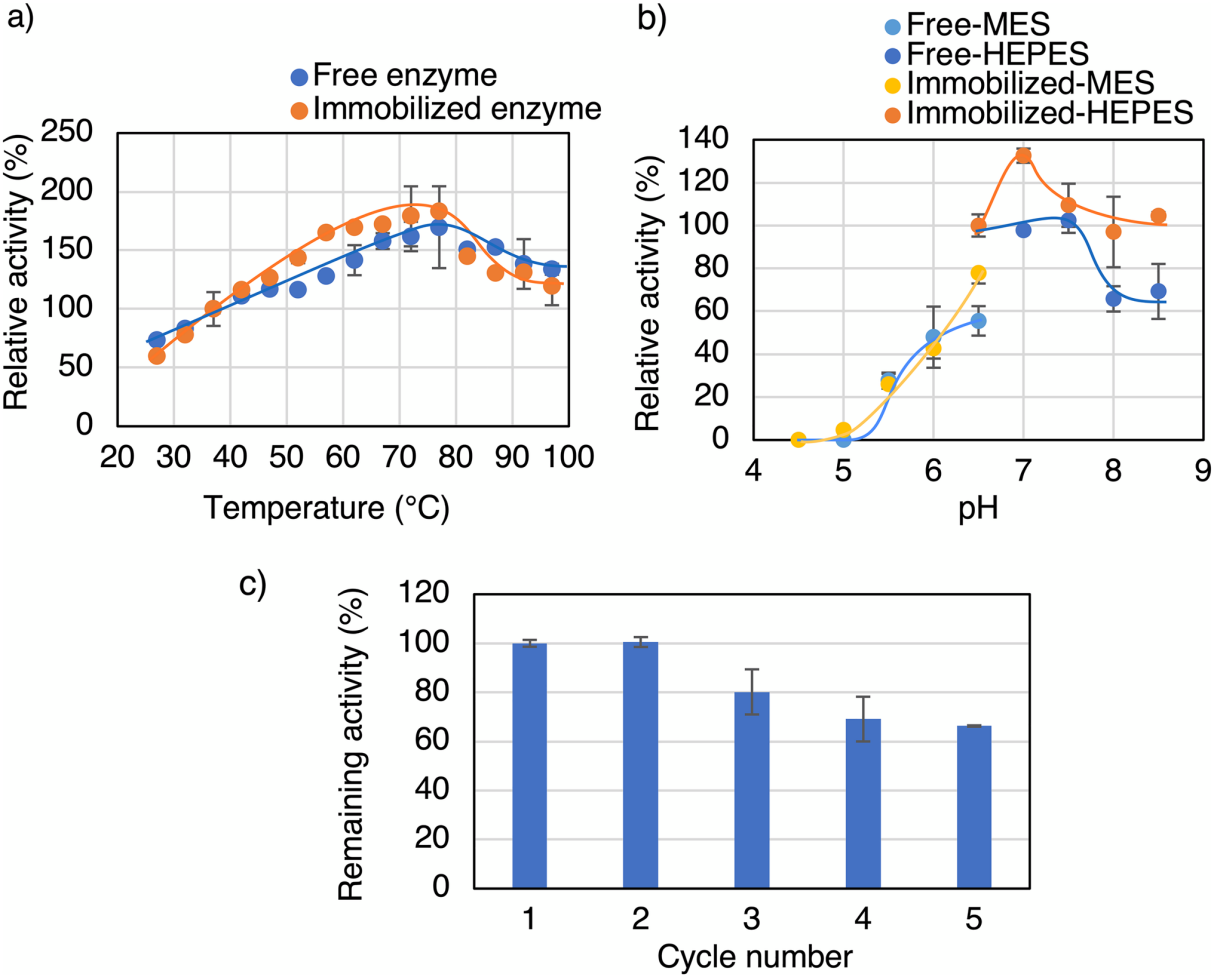
Effect of **a** temperature and **b** pH on the activity of free and immobilized *Ta*IDH, and **c** recyclability of immobilized *Ta*IDH. The activity below 77 °C was slightly higher for the immobilized enzyme than for the free enzyme, but that above 82 °C was opposite. **a** *t* test to analyze the higher activity of the immobilized enzyme than the free enzyme at 57–67 °C: *P*-value (one-sided test) of 57 °C: 0.000028, 62 °C: 0.084, 67 °C: 0.093) There was no significant effect of immobilization on optimum pH. **b** *t* test to analyze the higher activity of the immobilized enzyme than the free enzyme at alkaline region: *P*-value (one-sided test) of pH 7.0: 0.0045, pH 7.5: 0.31, pH 8.0: 0.11, pH 8.5:0.056) The immobilized *Ta*IDH could be used up to five times, with a remaining activity of 66%

The immobilized *Ta*IDH synthesized under the optimal conditions (pH 7.0 and 7.5 mM PBS, 25 mM Mn^2+^, and 0.05 mg protein/mL) was characterized and compared with the free enzyme, and the morphology was examined by SEM analysis (Fig. S2c). Examining the effect of temperature on the oxidative decarboxylation activity of free and immobilized *Ta*IDH revealed that the optimum temperature was 77 °C for both. The activity below 77 °C was slightly higher for the immobilized enzyme than for the free enzyme (Fig. 5a). Examination of the effect of pH on the oxidative decarboxylation activity of free and immobilized *Ta*GDH revealed that their activities were similar under acidic conditions. In contrast, the activity of the immobilized enzyme was slightly higher than that of the free enzyme under alkaline conditions (pH > 7.0) (Fig. 5b). The recyclability of the *Ta*IDH nanocrystals was subsequently examined. It was found that the *Ta*IDH nanocrystal could be used up to five times, with a remaining activity of 66% (Fig. 5c).

### Carboxylation by Immobilized *Ta*IDH and *Ta*GDH

Immobilized enzymes were employed to the carboxylation of 2-ketoglutaric acid to produce isocitric acid directly using CO_2_ as a substrate. The immobilized enzymes (*Ta*IDH: 0.50 U, *Ta*GDH: 0.032 U), NADPH, MnCl_2_, 2-ketoglutaric acid (20 mM), d-glucose as an auxiliary substrate for coenzyme recycling, and HEPES buffer were added to a pressure-resistant vessel. CO_2_ was introduced to 1.0 MPa, and the solution was stirred for 30 min at 37 °C. After quenching the reaction, the yield of the isocitrate was determined, resulting in 24%.

## Discussions

Firstly, *Ta*GDH and *Ta*IDH were successfully immobilized using Mn^2+^. The investigation on the type of metal for *Ta*GDH immobilization revealed that the suitable metals are Mn^2+^ and Co^2+^, but no precipitation occurred using Ca^2+^ and Mg^2+^. The difference between metal types may depend on the affinity of metal ions for histidine and cysteine residues of enzymes [24]. It is likely that Co^2+^ forms a precipitate because it can bind to the His tag of the protein, as in *G. candidum* acetophenone reductase [15]. However, because the metal type that forms precipitates largely depends on the protein [7, 8], as shown in Table S3, systematic studies to elucidate the relationship between the precipitate-forming metal ion types and the surface structures of the protein are necessary.

After the selection of the suitable metal ion, the remaining activity was investigated, and it was found that the immobilization process enhanced *Ta*IDH activity up to 211% of its original activity at 25 mM Mn^2+^ (Fig. 4b), while *Ta*GDH retained up to 71% at 5 mM Mn^2+^ (Fig. 2a), and up to 48% at 25 mM (Fig. 2b). The significant increase in *Ta*IDH activity after immobilization is notable. This suggests that the immobilization process not only preserved enzyme activity but also promoted overactivity. A plausible reason for the improved activity of *Ta*IDH may be the favorable interaction between the enzyme and the metal, as *Ta*IDH requires a divalent caution for its activity [22]. Further studies to determine the protein conformation of the immobilized *Ta*IDH are necessary to clarify the mechanism. Some enzyme-inorganic nanocrystals have also been reported to exhibit higher activity than free enzymes. For example, *Psychrobacter* sp. ZY124 lipase Z12-calcium phosphate nanocrystals [11], papain-Cu_3_(PO_4_)_2_ hybrid nanoflowers [25], *Geotrichum candidum* aldehyde dehydrogenase-Mn_3_(PO_4_)_2_ nanocrystals [16], etc. This is in contrast to the fact that enzymes immobilized by conventional methods generally have higher stability but lower activity than the free enzymes before immobilization [4]. The mild immobilization conditions of the nanocrystal method without using strong reagents such as glutaraldehyde may also contribute to preventing enzyme denaturation during the immobilization process [26]. The improved activity can also be attributed to the high porosity of the surface of the *Ta*IDH nanocrystals, as can be seen in the SEM images (Fig. S2). Given the large surface area, the transportation of substrates and products may not be as limited as in conventional immobilization methods [27]. Because all the nanocrystals with *Ta*GDH (Fig. S2b) and *Ta*IDH (Fig. S2c), and without the enzyme (control) (Fig. S2a) have porous surfaces, the presence of protein during the crystal formation may not be related to pore formation. Although the surface of *Ta*GDH nanocrystals also contained pores, an increase in activity was not observed. Therefore, the merit of having a highly porous structure may be canceled out by other factors in the case of *Ta*GDH. For the case using 5.0 mM Mn^2+^ (Fig. 2a), the immobilization yield and remaining activity are almost the same, which implies that the activity loss is mostly from the loss of protein. For the case of using 25 mM Mn^2+^(Fig. 2b), the immobilization yield is much higher than the remaining activity, which implies that the protein might be too tightly bound to keep the activity.

Then, the immobilized *Ta*GDH and *Ta*IDH are characterized by investigating the optimum temperature for activities and stabilities as the merits of the immobilization of enzymes are improvements in these aspects. Examination of the effect of temperature on the activity of free and immobilized revealed that the optimum temperatures were not affected by the immobilization for both *Ta*GDH (Fig. 3a) and *Ta*IDH (Fig. 5a). On the other hand, the immobilization improved the activity of *Ta*GDH in the high-temperature region slightly, possibly because it helped the enzyme maintain its proper structure. It is a great asset, especially for industrial applications where extreme conditions are often encountered. However, regarding *Ta*IDH, the immobilization slightly improved the activity below 77 °C. This is the opposite phenomenon from conventional observations, where immobilization usually improves stability but not activity [4].

One of the primary advantages of immobilized enzymes over free enzymes is their recyclability. As expected, both immobilized *Ta*GDH and *Ta*IDH were successfully reused with similar remaining activities in the 5th cycle (*Ta*GDH: 70% (Fig. 3c), *Ta*IDH: 66% (Fig. 5c)), despite the higher Mn^2+^concentration (25 mM) and lower protein concentration (0.05 mg protein/mL) for *Ta*IDH immobilization than those for *Ta*GDH, thus confirming the effectiveness of the nanocrystal immobilization method for *Ta*GDH and *Ta*IDH. Then, these immobilized enzymes were compared with other GDHs and IDHs in the literature (Table S4 and S5). The immobilization yield and remaining activity of the immobilized *Ta*GDH are higher than some of the methods, but reusability was similar or lower. However, this method has the advantage in the simpleness in the preparation of the immobilized enzyme, just mixing the metal and enzyme solutions, without the need for support. On the other hand, immobilized *Ta*IDH has a higher immobilization yield and remaining activities while reusability is higher or lower, depending on the methods. With these advantages and disadvantages, immobilized *Ta*GDH and *Ta*IDH, indicating potential for further applications, were used as carboxylation catalysts, next.

The carboxylation of 2-ketoglutaric acid to produce isocitric acid catalyzed by the immobilized *Ta*IDH and *Ta*GDH directly using CO_2_ as a substrate was performed. The success of carboxylation using CO_2_ is noteworthy since most of the previously-reported synthetic enzyme-catalyzed CO_2_ fixation reactions [28] such as reactions catalyzed by salicylic acid decarboxylase [29] and pyrrole-2-carboxylate catalyzed reaction [30–32] utilized carbonates such as KHCO_3_ as the source of carbon dioxide.

## Conclusions

To the best of our knowledge, this is the first study to immobilize GDH and IDH, crucial enzymes for coenzyme recycling and carboxylation, by forming enzyme-inorganic hybrid nanocrystals. The immobilization process led to an increase in *Ta*IDH activity, and the recycling of immobilized *Ta*GDH and *Ta*IDH was successful. In future studies, our focus will be the investigation of the mechanism of the improvement by immobilization, improvement in the shelf life of the immobilized enzymes, mutagenesis of the carboxylation enzyme to expand substrate specificity, and the development of flow carboxylation processes using the immobilized biocatalyst for scalable applications.

### Supplementary Information

Below is the link to the electronic supplementary material.

Supplementary file1 (PDF 1942 KB)

## Data Availability

Raw data/original images are available to be provided as supporting material upon request.
