# Supplementary material for: Immobilization of Thermoplasma acidophilum Glucose Dehydrogenase and Isocitrate Dehydrogenase Through Enzyme-Inorganic Hybrid Nanocrystal Formation
Source: Curr Microbiol. 2024 Jan 18;81(2):67. doi: 10.1007/s00284-023-03577-6 (PMC10796475; doi:10.1007/s00284-023-03577-6)
Supplement: Supplementary file 1 — Supplementary file1 (PDF 1942 KB) [file 284_2023_3577_MOESM1_ESM.pdf]

Supplementary information

Immobilization of *Thermoplasma acidophilum* glucose dehydrogenase and isocitrate dehydrogenase through enzyme-inorganic hybrid nanocrystal formation

Shusuke Oshima, Yuri Oku, Kotchakorn T.sriwong, Yutaro Kimura, Tomoko Matsuda

Department of Life Science and Technology, School of Life Science and Technology, Tokyo Institute of Technology
4259 Nagatsuta-cho, Midori-ku, Yokohama, 226-8501, JAPAN.

List of content

Experimental conditions to investigate the properties of the immobilized enzyme 1
Fig. S1 Reductive carboxylation of 2-ketoglutaric acid using CO_2 with *Thermoplasma acidophilum* isocitrate dehydrogenase (*Ta*IDH) and *T. acidophilum* glucose dehydrogenase (*Ta*GDH). 2
Fig. S2 SEM images of **a**) $Mn_3(PO_4)_2$ crystal (control), **b**) *Ta*GDH nanocrystal, and **c**) *Ta*IDH nanocrystal. 2
Table S1 Immobilization conditions. 2
Table S2 Effect of metals on free *Ta*GDH activity. 3
Table S3 Examples of His-tagged enzyme immobilization using Ca^{2+}, Mn^{2+}, Co^{2+}, and Mg^{2+}. 3
Table S4 Examples of immobilization of glucose dehydrogenases (GDHs). 4
Table S5 Examples of immobilization of isocitrate dehydrogenases (IDHs). 4
References 5

Experimental conditions to investigate the properties of the immobilized enzyme

Fig. 3a: 10 µL D-glucose (100 mM), 960 µL HEPES-NaOH buffer (100 mM, pH 6.5), and 10 µL purified or immobilized enzyme solution (1 U/mL) were incubated at 27-97 °C for 15 min. 1 mL scale assays were performed by adding 20 µL $NADP^+$ (10 mM).
Fig. 3b: 10 µL d-glucose (100 mM), 960 µl HEPES-NaOH buffer (100 mM) or MES-NaOH buffer (100 mM), and 10 µL purified enzyme solution (1 U/mL) were incubated at 37 °C for 15 min. 1 mL scale assays were performed by adding 20 µL $NADP^+$ (10 mM).
Fig. 3c: 1.5 mL scale assays were performed using $NADP^+$ (10 mM), d-glucose (10 mM) in HEPES-NaOH buffer (100 mM, pH 6.5), and immobilized *Ta*GDH (0.5 U) at 37 °C for 5 min. The catalyst was separated from the reaction mixture by centrifugation at 12,000 rpm for 5 min at 4 °C, washed with DW, and used for the next cycle.
Fig. 5a: 10 µL isocitric acid (10 mM), 20 µL $MgCl_2$ (20 mM), 940 µL HEPES-NaOH buffer (100 mM, pH 6.5), and 10 µL purified or immobilized enzyme solution (1 U/mL) were incubated at 27–97 °C for 15 min. 1 mL scale assays were performed by adding 20 µL $NADP^+$ (10 mM).
Fig. 5b: 10 µL isocitric acid (10 mM), 20 µl $MgCl_2$ (20 mM), 940 µl HEPES-NaOH buffer (100 mM) or MES-NaOH buffer (100 mM), and 10 µL purified or immobilized enzyme solution (1 U/mL) were incubated at 37 °C for 15 min. 1 mL scale assays were performed by adding 20 µl $NADP^+$ (10 mM).
Fig. 5c: 1.5 mL scale assays were performed using $NADP^+$ (10 mM), isocitric acid (10 mM), $MgCl_2$ (0.4 mM) in HEPES-NaOH buffer (100 mM, pH 6.5), and *Ta*IDH nanocrystal (0.5 U) at 37 °C for 5 min. The catalyst was then separated from the reaction mixture by centrifugation at 12,000 rpm for 5 min at 4 °C, washed with DW, and used for the next cycle.

The relative activity was based on the activity at 37 °C for Fig. 3a and Fig. 5a, at pH 6.5 MES-NaOH buffer for Fig. 3b, and at pH 6.5 HEPES-NaOH buffer for Fig. 5b as 100%. The activity during the first cycle was set at 100% for Fig. 3c and Fig. 5c.

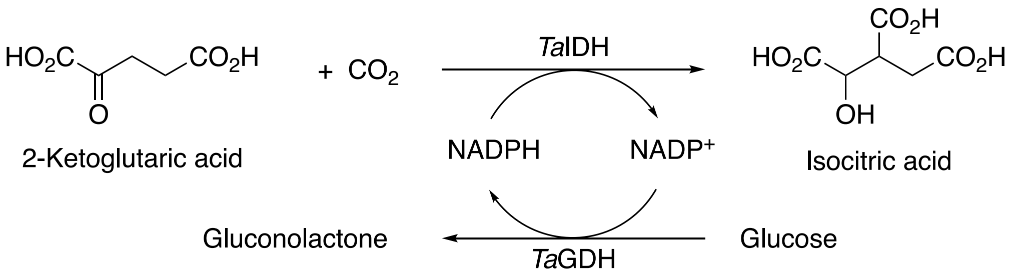

Fig. S1 Reductive carboxylation of 2-ketoglutaric acid using CO_2 with *Thermoplasma acidophilum* isocitrate dehydrogenase (*Ta*IDH) and *T. acidophilum* glucose dehydrogenase (*Ta*GDH). The carboxylation is accelerated by coupling the *Ta*GDH-catalyzed NADPH regeneration.

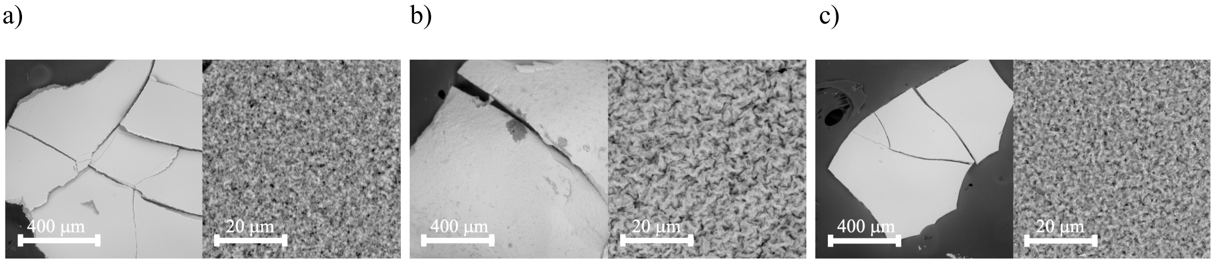

Fig. S2 SEM images of **a**) $Mn_3(PO_4)_2$ crystal (control), **b**) *Ta*GDH nanocrystal, and **c**) *Ta*IDH nanocrystal.

Table S1 Immobilization conditions

	Enzyme immobilized	Metal ion	pH	PBS conc. (mM)	Metal ion conc. (mM)	Protein conc. (mg/ml)	Activity measurement[a,b]
Fig. 1a	*Ta*GDH	Varied	7.0	7.5	5.0	0.50	A
Fig. 1b	*Ta*GDH	Mn^{2+}	Varied	7.5	5.0	0.50	A
Fig. 1c	*Ta*GDH	Mn^{2+}	7.0	Varied	5.0	0.50	A
Fig. 1d	*Ta*GDH	Mn^{2+}	7.0	7.5	Varied	0.50	A
Fig. 2a	*Ta*GDH	Mn^{2+}	7.0	7.5	5.0	Varied	A
Fig. 2b	*Ta*GDH	Mn^{2+}	7.0	7.5	25	Varied	A
Fig. 4a	*Ta*IDH	Mn^{2+}	7.0	7.5	Varied	0.50	B
Fig. 4b	*Ta*IDH	Mn^{2+}	7.0	7.5	25	Varied	B

[a]A: 37 °C using 20 µl $NADP^+$ (10 mM), 10 µl D-glucose (100 mM) in 960 µl HEPES-NaOH buffer (100 mM, pH 6.5) and 10 µl immobilized enzyme solution (0.25 mg/ml). The remaining activity was based on the activity of free *Ta*GDH in 0.10 M HEPES-NaOH buffer pH 6.5 as 100%.

[a]B: 37 °C using 20 µl $NADP^+$ (10 mM), 10 µl isocitric acid (10 mM), 20 µl $MgCl_2$ (20 mM) in 940 µl HEPES-NaOH buffer (100 mM, pH 6.5) and 10 µl immobilized enzyme solution (0.1 mg/ml). The remaining activity was based on the activity of free *Ta*IDH in 0.10 M HEPES-NaOH buffer pH 6.5 as 100%.

Table S2 Effect of metals on free *Ta*GDH activity.

Metal ion	Conc. (mM)	Relative activity (%)
None	-	100
$MnCl_2 \cdot 4H_2O$	1.0	103.2±10.9
$ZnSO_4 \cdot 7H_2O$	0.2	48.1±4.7
$MgSO_4 \cdot 7H_2O$	1.0	102.2±6.1
$CuSO_4 \cdot 5H_2O$	0.02	70.5±7.9
$CaCl_2 \cdot 2H_2O$	1.0	104.3±2.7
$CoCl_2 \cdot 6H_2O$	1.0	80.2±4.4
$NiSO_4 \cdot 6H_2O$	1.0	54.2±8.6
$FeCl_3$	0.1	75.6±5.1
KCl	1.0	80.5±2.0
NaCl	1.0	94.6±2.6

10 µl D-glucose (100 mM), 960 µl HEPES-NaOH buffer (100 mM, pH 6.5) containing metal, and 10 µl purified enzyme solution (1 U/ml) were incubated at 37 °C for 15 min. Then, 1 mL scale assays were done by adding 20 µl $NADP^+$ (10 mM) at 37 °C. Oxidation activity was determined by the increase of NADPH absorbance at 340 nm. Relative activity was based on the activity without any metal ions as 100%.

Table S3 Examples of His-tagged enzyme immobilization using Ca^{2+}, Mn^{2+}, Co^{2+}, and Mg^{2+}.

Enzyme	Ca^{2+}	Mn^{2+}	Co^{2+}	Mg^{2+}
*Ta*GDH (This study)	-	+++	+++	-
Geotrichum candidum acetophenone reductase [1]	-	+	+++	-
Geotrichum candidum aldehyde dehydrogenase [2]	++	+++	+	n.d.
Fusarium sp. Baeyer-Villiger monooxygenase [3]	+++	+	++	+++

Examples of His-tagged enzyme immobilization using Ca^{2+}, Mn^{2+}, Co^{2+}, and Mg^{2+} by the same procedure with this study are listed. There are reviews summarizing the effect of metal types on immobilization by the method of forming enzyme-inorganic hybrid nanocrystal [4–6].

+++ Most suitable metal among metals listed in this Table.

++ Suitable metal.

+ Less suitable metal.

- No precipitate or a very small amount of precipitate formation.

n.d. not determined.

Table S4 Examples of immobilization of glucose dehydrogenases (GDHs)

Enzyme	Support/method	Immobilization yield (%)	Remaining activity (%)	Reusability	Reference
Thermoplasma acidophilum GDH *Ta*0897	Enzyme-inorganic hybrid nanocrystals	72	71	70% in the 5th cycle (Fig. 3c)	This work
Bacillus megaterium GDH	Aminopropyl controlled-pore silica	-	0.06~1.18	Reused 12 times without total loss of activity	[7]
Bacillus megaterium GDH	DEAE-Sephadex	-	30.67~42.67	Maintained their activities after 10 cycles	[7]
Bacillus megaterium GDH	Aldehyde functional ReSyn™ polymer microspheres	-	41.5	-	[8]
GDH-01[a]	Methacrylate/Styrene resin	40.8~100	21.3~28	-	[9]
GDH-01[a]	Monoaminoethyl-N-aminoethyl (MANA)-agarose	98.4 ± 0.2	105.5 ± 3.8	-	[9]
Bacillus sp. GDH	Graphene oxide magnetic nanoparticles	92	91	88% after 10 cycles	[10]

[a]Supplied by InnoSyn B.V. (Geleen, The Netherlands).

Table S5 Examples of immobilization of isocitrate dehydrogenases (IDHs)

Enzyme	Reaction	Support / method	Immobilization yield (%)	Remaining activity (%)	Reusability	Reference
Thermoplasma acidophilum IDH *Ta*0117	Decarboxylation	Enzyme-inorganic hybrid nanocrystals	>99	211	66% in the 5th cycle (Fig. 5c)	This work
Azotobacter vinelandii IDH	Decarboxylation	Sepharose 4B	60~75	30~40	-	[11]
Porcine heart IDH	Carboxylation	Mesoporous silica foam	93.6	15.1	About 45% after 10 cycles	[12]
IDH type I[a]	Decarboxylation	Magnetic nanoparticles (MNPs) modified by hyaluronic acid (HA) (Cross-linked enzyme aggregate (CLEAs))	-	93.4	80% after 15 cycles	[13]
IDH type I[a]	Decarboxylation	MNPs modified by HA and bovine serum albumin (BSA) (CLEAs)	-	98.9	85% after 15 cycles	[13]

[a]Supplied by BIMAS-RC (Sakarya, Turkey).

References

1. T.sriwong K, Koesoema AA, Matsuda T (2020) Organic-inorganic nanocrystal reductase to promote green asymmetric synthesis. RSC Adv 10:30953–30960. https://doi.org/10.1039/d0ra03160g
2. T.sriwong K, Ogura K, Hawari MA, Matsuda T (2021) Geotrichum candidum aldehyde dehydrogenase-inorganic nanocrystal with enhanced activity. Enzyme Microb Technol 150:109866. https://doi.org/10.1016/j.enzmictec.2021.109866
3. Takagi M, T Sriwong K, Masuda A, et al (2022) Immobilization of Baeyer-Villiger monooxygenase from acetone grown Fusarium sp. Biotechnol Lett 44:461–471. https://doi.org/10.1007/s10529-022-03224-3
4. Cui J, Jia S (2017) Organic–inorganic hybrid nanoflowers: A novel host platform for immobilizing biomolecules. Coord Chem Rev 352:249–263. https://doi.org/10.1016/j.ccr.2017.09.008
5. Tran TD, Kim M Il (2018) Organic-Inorganic Hybrid Nanoflowers as Potent Materials for Biosensing and Biocatalytic Applications. Biochip J 12:268–279. https://doi.org/10.1007/s13206-018-2409-7
6. T.sriwong K, Matsuda T (2022) Recent advances in enzyme immobilization utilizing nanotechnology for biocatalysis. Org Process Res Dev 26:1857–1877. https://doi.org/10.1021/acs.oprd.1c00404
7. Baron M (1997) Stabilization and reutilization of bacillus megaterium glucose dehydrogenase by immobilization. Appl Biochem Biotechnol - Part A Enzym Eng Biotechnol 63–65:257–268. https://doi.org/10.1007/BF02920429
8. Twala B V., Sewell BT, Jordaan J (2012) Immobilisation and characterisation of biocatalytic co-factor recycling enzymes, glucose dehydrogenase and NADH oxidase, on aldehyde functional ReSyn^{TM} polymer microspheres. Enzyme Microb Technol 50:331–336. https://doi.org/10.1016/j.enzmictec.2012.03.003
9. Solé J, Brummund J, Caminal G, et al (2019) Trimethyl-ε-caprolactone synthesis with a novel immobilized glucose dehydrogenase and an immobilized thermostable cyclohexanone monooxygenase. Appl Catal A Gen 585:117187. https://doi.org/10.1016/j.apcata.2019.117187
10. Wang J, Zhao M, Lu X, et al (2021) Covalent immobilization of glucose dehydrogenase onto graphene oxide magnetic nanoparticles to improve the stability. Brazilian J Chem Eng 38:265–272. https://doi.org/10.1007/s43153-021-00102-9
11. Chung AE (1972) Immobilized isocitrate dehydrogenase: Some aspects of its catalytic, chemical and immunological properties. Arch Biochem Biophys 152:125–135. https://doi.org/10.1016/0003-9861(72)90200-7
12. Xia S, Zhang L, Veony E (2019) Immobilization of Isocitrate Dehydrogenase on Mesoporous Silica Foam for Carbon Dioxide Capture. E3S Web Conf 93:1–5. https://doi.org/10.1051/e3sconf/20199304001
13. Farhan LO, Mehdi WA, Taha EM, et al (2021) Various type immobilizations of Isocitrate dehydrogenases enzyme on hyaluronic acid modified magnetic nanoparticles as stable biocatalysts. Int J Biol Macromol 182:217–227. https://doi.org/10.1016/j.ijbiomac.2021.04.026
